# Usutu virus: An emerging flavivirus with potential threat to public health in Africa: Nigeria as a case study

**DOI:** 10.3389/fvets.2023.1115501

**Published:** 2023-02-16

**Authors:** Olalekan Chris Akinsulie, Ridwan Olamilekan Adesola, Adetolase Bakre, Oluwawemimo Oluseun Adebowale, Richard Adeleke, Seto Charles Ogunleye, Ifeoluwa Peace Oladapo

**Affiliations:** ^1^College of Veterinary Medicine, Washington State University, Pullman, WA, United States; ^2^Faculty of Veterinary Medicine, University of Ibadan, Ibadan, Oyo State, Nigeria; ^3^College of Veterinary Medicine, Federal University of Agriculture, Abeokuta, Nigeria; ^4^College of Veterinary Medicine, Cornell University, Ithaca, NY, United States; ^5^College of Veterinary Medicine, Mississippi State University, Starkville, MS, United States; ^6^Department of Veterinary Parasitology, Universidade Federal de Parana, Curitiba, Brazil

**Keywords:** Usutu virus, Culex mosquitoes, migratory birds, flavivirus, public health

## Abstract

Usutu virus (USUV) is an arthropod-borne virus (arbovirus) of the flaviviridae family (genus *Flavivirus*) which belong to the Japanese encephalitis virus complex. Culex mosquitoes have been implicated in the transmission of this pathogen. The major susceptible hosts of USUV are migratory birds, thereby potentiating its ability to spread from one region to another globally. Nigeria has the largest economy in Africa with a significant percentage of the gross domestic product relying on the agricultural and animal production industry. This review explores the zoonotic potentials of the virus in Africa, especially Nigeria, with special focus on the devastating sequelae this might lead to in the future if necessary precautionary policies are not enacted and adopted to bolster the surveillance system for mosquito-borne viruses.

## Introduction

**Figure 1:**
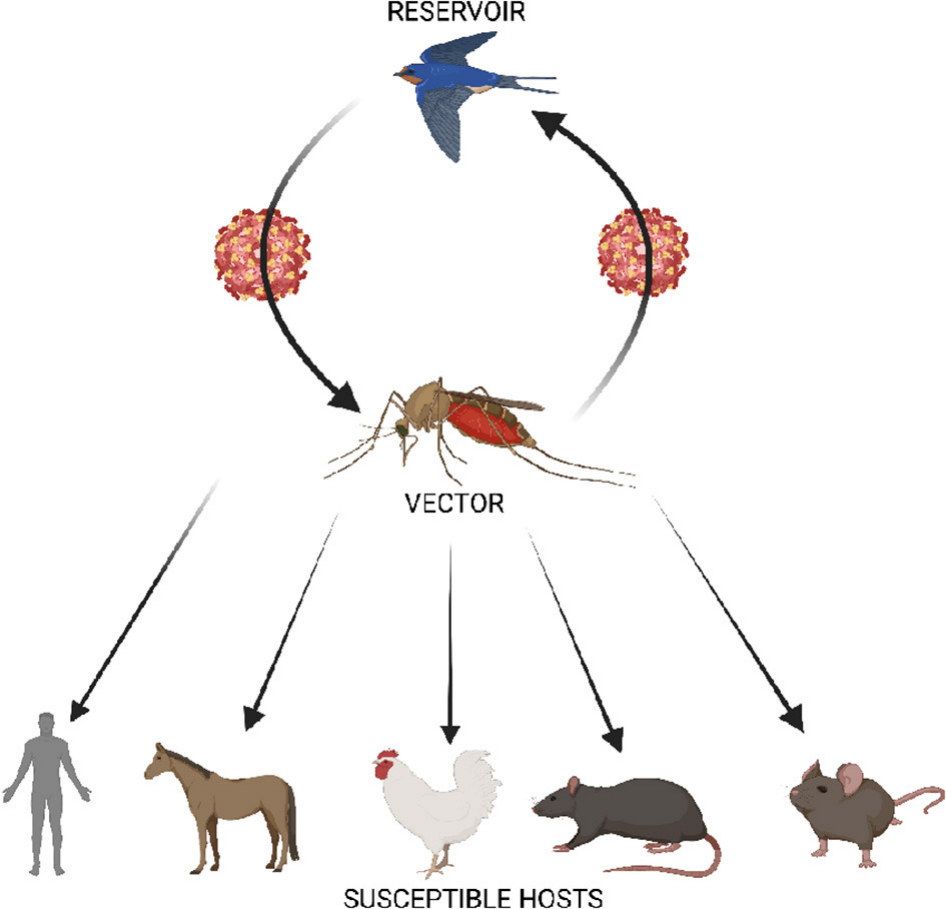
Schematic representation of the transmission cycle of USUV showing the enzootic cycle between *Culex* mosquito vector and birds, and the biological transmission of the virus to susceptible hosts. Humans have been identified as dead end host. Serological evidence of USUV infection has been reported in horse and avian population in Slovakia and Poland, and in African countries such as Central African Republic, Cote d'lvoire, Senegal Nigeria, and Uganda (2). In addition, USUV has been found in other animals including dogs, bats, red deer, rodents (shrews) (3).

**Figure 2:**
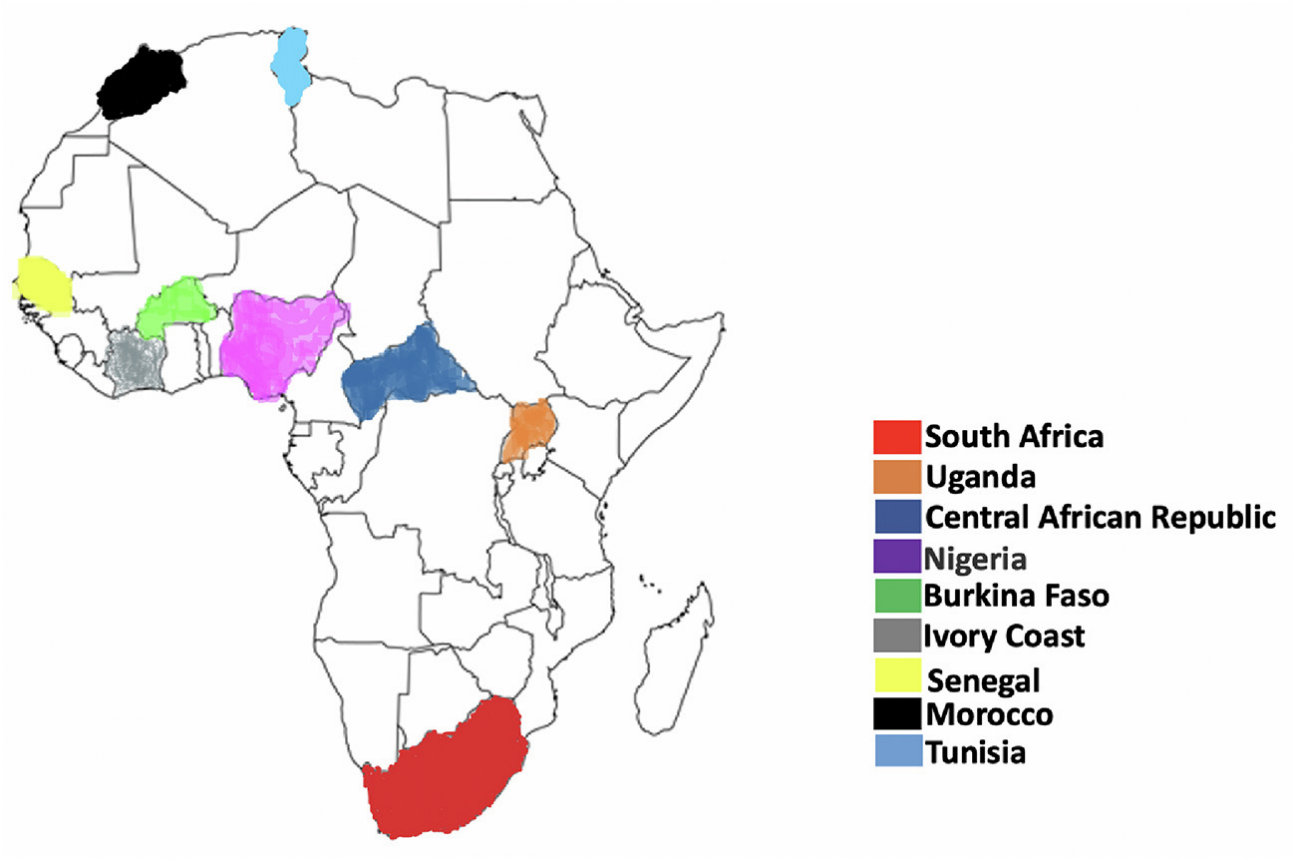
Geographical representation of Usutu virus distribution in Africa. The map represents countries where there have been reported presence of USUV. As depicted here, USUV has been detected at least once in Burkina Faso, Central African Republic, Ivory Coast, Morocco, Nigeria, Senegal, Tunisia, and Uganda. There have been reported presence of USUV also in Kenya, Madagascar, Mali, and Swaziland. Adapted from Clé et al. (1).

Usutu virus (USUV) previously restricted to sub-Saharan Africa, is now considered an emerging arboviral pathogen attracting the attention of the scientific and public health community due to its potential to spread to USUV-free areas, cause substantial mortalities in several avian species worldwide and consequential zoonotic impact on humans (1). USUV is an arbovirus of the flaviviridae family (genus Flavivirus) in the Japanese encephalitis virus complex. Other related viruses in this complex includes the Japanese encephalitis virus (JEV), West Nile virus (WNV), Yellow fever virus, Dengue and Murray Valley encephalitis virus (MVEV) which are some of the most pathogenic arboviruses of health impact to humans and animals (1). USUV was first discovered from mosquitoes in South Africa in 1959 and has since been reported in animal (rats, horses and dogs) and human hosts (Figure 1) in African regions like South Africa (4), West Africa (2), Central African Republic (1), Northern Africa (5), and East Africa (6) (Figure 2). Also, USUV has spread from Africa to Europe (emerged in the continent in 2001) and been documented in wide range of animals (birds, boars, squirrels, chimpanzees, reptiles, or horses) and humans in member countries such as Germany (7), Czech Republic (8), Hungary (9), Italy (10), Portugal (11), Spain (12), Austria (13), and the United Kingdom (14). The natural transmission cycle of USUV involves mosquitoes (*Culex* and to a lesser extent *Aedes* vectors) and birds that serve as amplifying hosts. Humans and other mammals are considered incidental (“dead-end”) hosts to this virus (15). The emergence and increase of USUV in wild birds, mosquitoes and humans shows the virus may now be endemic in several European countries as in African nations and may become a potential global health threat in the future. It is interesting to know that despite being a virus originating from Africa, little information still exists about the epidemiology, ecology, transmission dynamics and cycle of the virus, and the zoonotic potential and burden in humans in Africa.

## Genetic diversity of Usutu virus in Africa

**Figure 3:**
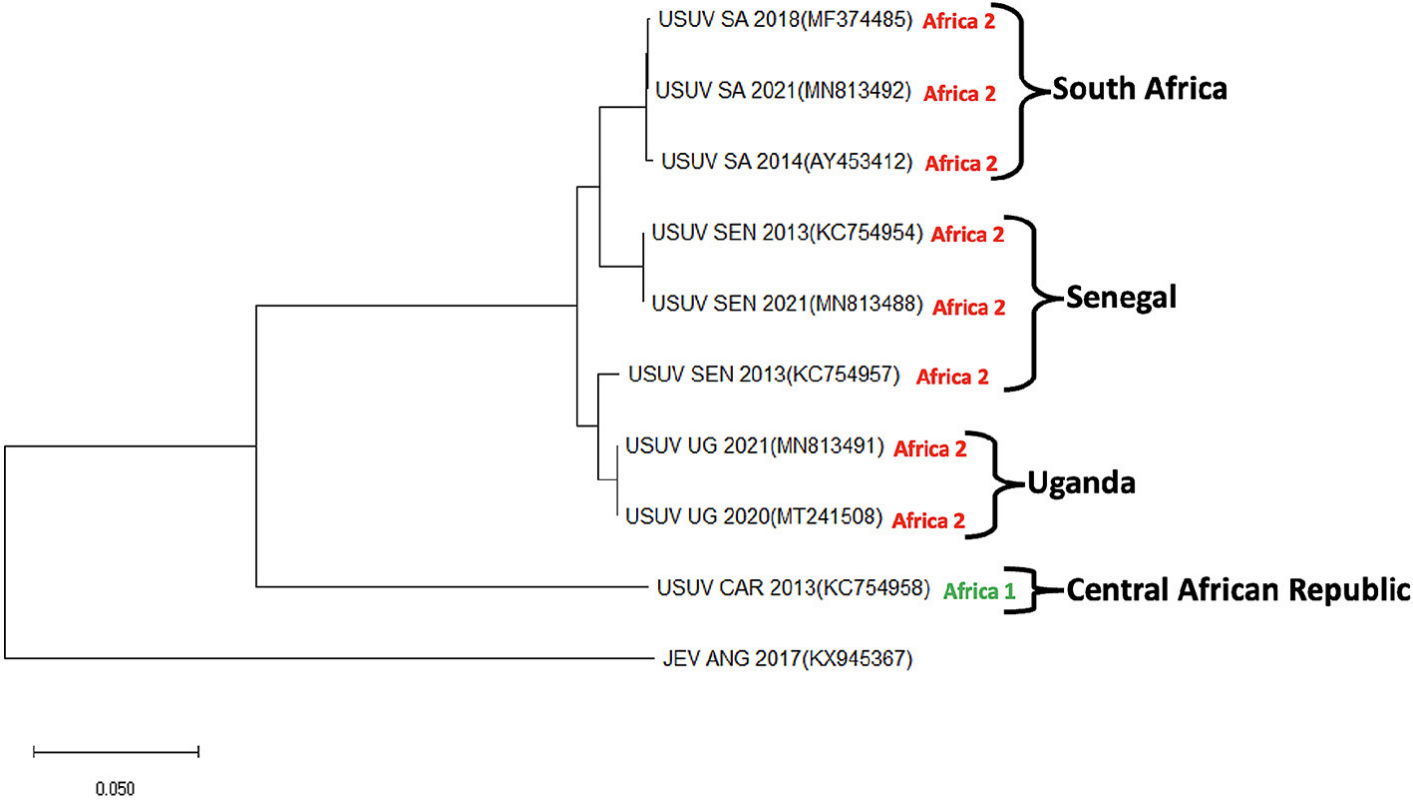
Phylogenetic tree showing Usutu virus strains isolated in 4 African countries (South Africa, Senegal, Uganda, and Central African Republic). USUV strains isolated from Africa can be classified into three clades (I–III). Sequences for Clade III strains specifically isolated from Africa are not currently available and thus not included in this tree. The evolutionary history was deduced using the maximum Likelihood method and Tamura-Nei model. Evolutionary analyses were conducted in the MEGA-X. This analysis involved 10 nucleotide sequences. The sequences were derived from Genbank repository with accession numbers: Usutu virus strains (USUV); MF374485, MN813492, AY453412, KC754954, MN813488, KC754957, MN813491, MT241508, and KC754958 and Japanese encephalitis virus (JEV); KX945367. JEV serves as an outgroup.

As related to other flaviviruses, USUV has a (+)-strand RNA genome of 11,064 nucleotides that encodes a single polyprotein of 3,434 amino acids that is consequently sliced into structural (C, E, and prM) and non-structural (NS1, NS2A, NS2B, NS3, NS4A, NS4B, and NS5) proteins (16). USUV is categorized into three (2) lineages which are diverse in African countries, based on the geographic origin of isolation (Figure 3). A phylogenetic study of the Nonstructural protein 5 (NS5) gene confirmed that USUV strains from Africa belong to 3 discrete lineages (Africa I-III) (17). The Africa lineage I comprise only one strain (CAR-1969 strain) isolated in 1969 in the Central African Republic. Also, the Africa II lineage investigated in South Africa in the mid-1940's includes USUV strains isolated in Senegal (16). Furthermore, the Africa III lineage comprises strains isolated in Senegal and a human isolate from the Central African Republic isolated in 1981. Currently, there are few whole genome sequences of USUV strains available from Africa. Future research on USUV involving the phylogenetic analysis of USUV strains isolated from African birds would bolster the understanding of the virus dispersal between Africa and Europe (4). According to Cadar et al. (18), there is sequence identity across 94% of USUV complete genome sequences considered, with the exclusion of the CAR-1969 isolate which showed a genome identity of 78.3% related to other USUV strains. A comparison of untranslated regions (UTR) revealed that 5′ UTR conserves a comparable secondary structure and size among diverse lineages, with the exclusion of Africa I lineage. At the same time, specific nucleotide mutations are seen in 5′ UTR in lineages in African countries (A3T, T4C, C10T, and T14C) (19). In 3′ UTR, highly adjustable size heterogeneity is seen among different lineages. Relative genomic analysis between USUV proteins showed specific amino acid mutations associated with the geographical origin of hosts and isolation. Geographic-specific mutations observed in all African lineages are based on changes in the Non-structural protein 4B (M16I) and Capsid protein (A120V). In similar fashion, host-specific mutations have been seen in birds (Y120N, prM). Also, in humans, unique amino acid mutations were discovered in the CAR-1981 strain (NS2A, S154L; NS3 Y474H; NS5, H173Q), and the USUV strain isolated from patients in Africa with skin rashes.

## Vectors and co-circulation dynamics of Usutu virus

### Mosquito

The environment is a critical factor in the successful circulation of viral infections. USUV has been associated with multiple birds' deaths and has been linked to neurotropic symptoms in humans (20). *Culex* mosquitoes which are the main mosquitoes involved in transmitting USUV, Japanese Encephalitis virus, and West Nile virus, are ornithophilic, greatly contributing to birds' involvement in this virus transmission. In Africa, USUV has been isolated from *Culex neavei*, and *Culex univittatus* (21). Importantly, common *Culex* mosquitoes which have been identified in Nigeria such as *Culex perfuscu*, and *Culex quinquefasciatus*, a member of *Culex pipiens* complex, are also involved in the circulation of USUV to migratory birds (4). However, *Culex quinquefasciatus* is the most abundant in a domestic environment and the most anthropophilic among the mosquito vectors for USUV (4). The possible involvement of *Aedes albopictus*, which has also been identified predominantly in southeastern parts of Nigeria (22), in the transmission cycle of USUV was confirmed in a study conducted in Italy (10). This study showed the simultaneous circulation of both West Nile and USUV by *Aedes albopictus* mosquito. Overall, *Culex pipiens* is the most competent vector for USUV because *Aedes albopictus* shows a lower competence even though the virus can replicate in the Mosquito's gut (23). Currently, more investigations are ongoing to identify more competent vectors involved in the zoonotic transmission cycle of USUV and other flaviviruses in Nigeria and Africa at large. In North America, *Culex pipiens*, and *Culex quinquefasciatus* were identified as competent vectors in the transmission of USUV following the infection of these mosquito vectors (23) USUV detection was also confirmed in mosquito species such as: *Aedes japonicus, Aedes vexans, Anopheles maculipennis, Anopheles plumbeus, Coquillettidia richiardii, Culiseta annulata, and some Ochlerotatus species* (24). To date, no studies have confirmed the presence of USUV in ticks, even in regions with significant USUV circulation (24). Routine surveillance is important in monitoring the adaptation of USUV in new mosquito vectors. A single mutation was necessary to adapt Chikungunya virus in *Aedes albopictus* (25). This surveillance will aid the rapid detection of USUV circulation into new geographical settings (3).

### Birds

In Africa and Europe, birds act as the main natural and amplifying hosts in the transmission cycle of many pathogens including USUV. So far, the infection has been identified in over 93 different species of birds in thirty-five families (26). There is a correlation between birds' abundance and the circulation of USUV in mosquito vectors. In a survey carried out in Spain to screen mosquitoes for the presence of USUV genome, there was a link between passerine bird abundance and the presence of USUV in *Culex* vectors (9). In Africa and Europe, more than 60 avian species and migratory birds contribute to the circulation of USUV. Specifically in Nigeria, USUV was isolated from Kurrichane thrush (*Turdus libonyanus*), piping hornbill (*Bycanistes sharpei*), and little greenbull (*Andropadus virens)* in 1972 (27). Generally, the main clinical signs of USUV in infected birds include ataxia, anorexia, loss of weight, disorientation, and recumbency. The predominant gross pathologies include enlargement of the liver and spleen. Histologically, there is usually inflammatory infiltrates and necrotic foci in the spleen, heart, brain, and kidney of infected birds (28). In certain areas in Europe, such as Germany, France, and the Netherlands, USUV infection resulted in high mortality in the bird population. More studies aimed at investigating the mortality of birds and the basis for the variation in pathogenicity among different bird species in Nigeria and Africa is required.

### Humans

So far, USUV infection has a very low incidence in humans in Africa and Europe, where the virus is circulating in the Culex mosquito vectors. To date, only few human cases have been identified. In Africa, the first detection of USUV infection occurred in 1981 in the Central African Republic. The patient had a fever and body rash. The second case was identified in a patient from Burkina Faso in 2004 who had fever and jaundice (4). In humans, USUV infection is usually characterized by fever, headache, tremors in the hand and hyperreflexia. In Europe, the first human report of USUV infection was made in Italy in 2009. The patient had a preexisting B cell lymphoma tumor and had a fever and neurologic symptoms. Also, in 2009, a woman in Italy was diagnosed with a sickness related to USUV infection following a liver organ transplant because of thrombotic thrombocytopenic purpura that developed during an acute episode of USUV infection. The first two human cases of USUV associated neuroinvasive infection were confirmed in immunocompromised patients in Italy in 2009 (29). The case report by Cavrini et al. (29) confirmed USUV by heminested RT-PCR assay targeting the NS5 gene of flaviviruses. Also, a retrospective analysis of human samples (serum and CSF) collected between 2008 and 2011 with or without neurological impairments in Modena, Italy documented a seroprevalence of about 6.6% (30). Similarly, USUV was detected in birds and mosquitoes, and partial sequencing of the virus genome revealed high nucleotide sequence similarity within hosts and strains from Central Europe. In 2014 and 2016–2018, much higher prevalence of the virus (18.1 and 46.3%, respectively) was detected in forest workers and healthy blood donors by 29, suggesting a rise and threat to human health. In 2013 another three cases of USUV neuro-invasive infection (Europe lineage 2) was reported in Croatia during a WNV outbreak (31). Like the Italian report, two of the cases were immunocompromised with arterial hypertension, hyperlipidemia, and diabetes mellitus, thus indicating comorbidities may have a role in the pathogenicity of USUV. Patients presented meningitis and meningoencephalitis with clinical signs such as headache, fever, nuchal rigidity, hand tremor, hyperreflexia, and memory loss and speech fluency difficulty. Another study in Croatia in 2018 further detected three cases, one of the patients was immunocompromised with chronic lymphocytic leukemia and fatal meningoencephalitis (15). USUV has also been reported in animals such as bats in 2013 (32), wild and captive birds in 2011 (28), Owls (33) and humans (18) in Germany. A survey of wild birds in 2011 detected USUV in 38.6% in dead birds using real-time RT-PCR (28). These data showed that the virus spread and caused epizootics among wild and captive birds in south-west Germany in 2011. The incidence of the virus has then increased and over 1300 cases were detected since 2013. Furthermore, in January 2012, a total of 4,200 serum samples from healthy blood donors from south-west Germany were analyzed for USUV-specific antibodies. Although all serum samples from the patients tested negative, one person was confirmed with IgG- and IgM antibodies and USUV infection, after eliminating the possibility of serological cross-reactivity to other flaviviruses (34). Likewise, during the 2016 epizootics, a surveillance of WNV and USUV from 13, 032 blood donors confirmed the presence of USUV Europe 3 lineage in a blood donor, which later revealed an acute USUV infection by immunofluorescence, PCR and genome sequencing assays (18). USUV from the donor plasma showed 99% homology sequence with those found in the birds during the epidemic in 2016. Hungary is another European country that has documented the presence of USUV in animals and humans. The country reported the first incidence during the country's dead bird surveillance program in a black bird (*Turdus merula*) in August 2005 and further six birds in 2006 by reverse transcription-PCR, immunohistochemistry, in situ hybridization, viral isolation and whole genome sequencing. A total of 332 dead birds belonging to various species were tested for USUV infection between 2003 and 2006. The Hungary USUV strain shared 99.9% identity with the circulating Austrian strain indicating that the USUV strain responsible for the epidemic in blackbird in spread from Austria to Hungary (9). Moreover, results of passive surveillance of USUV in Austria and Hungary (between 2010 and 2016) revealed 12 birds tested positive for USUV infection in 2016 in Hungary (35). Similar pathological conditions as previously described by Bakonyi et al. (35) were reported. The pathological examinations showed moderately autolytic condition, emaciation, moderate to severe splenomegaly, mild hepatomegaly and general congestion of internal organs (Table 1). The nucleotide sequences of USUVs detected cluster together with sequences identified in a human plasma sample and in blackbirds between 2009 and 2010 in Italy. The first human case of USUV was reported by Nagy et al. (44) during a surveillance programme for WNV in 2018. Patients with clinical suspicion of acute WNV infection were tested in parallel for WNV, tick-borne encephalitis virus and Usutu virus (USUV) by serological methods. The Europe lineage 2 USUV infection was confirmed in 2018 in a patient with aseptic meningitis by serology and molecular assays. Furthermore, in 2019, a year after the largest WNV epidemic in Europe, a comprehensive sero survey for WNV and USUV was conducted among blood in Hungary to assess the exposure of the Hungarian population (44). A total of five blood donors were USUV seropositive out of 3,005 donors tested (0.17%). This shows an increase in human donors with possible clinical manifestation expected in Hungary (44). The first emergence of USUV European lineage 2 was reported in Austria in 2001 where it received wide public attention due to the epidemics and high mortality among Eurasian blackbirds (*Turdus merula*) and great gray owls (*Strix nebulosa*) in the Vienna (38). A 3-year nationwide surveillance of the USUV in dead wild birds demonstrated 92 birds (in 2003), 11 (in 2004) birds, and 4 (in 2005) birds positive to the infection. However, USUV associated avian deaths reduced during the course of the years due to the establishment of herd immunity in wild birds in Austria (52). Between 2010 and 2015, a few wild birds tested were all tested negative to the USUV infection (15). In 2017, USUV nucleic acid was detected in six of out of 12,047 blood donations from eastern Austria between July and August 2017. These detections were from healthy individuals (35). However, the first case of human USUV was recently reported by Graninger et al. (47). The patient, an 81-year-old man was presented to a hospital in Vienna, Austria in early September 2021. The patient was febrile and displayed confusion and difficulty in responding to questions. The patient later showed the intermittent clonic jerks of his right upper extremity. Subsequently, Serum and CSF samples were tested for USUV, and the CSF came out positive *via* USUV-specific RT-PCR (47). A partial sequence of the virus genome was 100% identical to sequences from humans, birds, and mosquitoes in the Czech Republic, Hungary, and Austria and confirmed as the Europe 2 lineage (47). Similarly, in France and the Netherlands, the first outbreak of USUV were obtained in 2015 (48) and 2016 (50), respectively, which involved number of case reports of disease-associated and unusual mortality in blackbirds. In the subsequent year (2016) in France, the first human case was presented and associated with atypical neurologic signs (49). In the Netherlands, from April –September 2018, plasma from blood donors in three Dutch provinces with significant USUV activity and blackbird population were collected and analyzed for USUV infection using molecular assays (51). Six of the 12,040 Dutch blood donations were positive.

**Table 1:** A summary of countries that have reported both animal and human cases (incidental hosts) of USUV with confirmed vectors being *Culex* and *Aedes* mosquitoes.

**References**	**Country**	**Year**	**Animals**	**Humans**	**Clinical presentations**	**Diagnostic method**
Nikolay et al. (4)	Central African Republic	1981	-	√	Fever and rash	-
Nikolay et al. (4)	Burkina Faso	2004	-	√	Fever and jaundice	-
Chevalier et al. (36)	Mali	2008	Domestic birds	-	-	ELISA, Micro neutralization Test
Chevalier et al. (36)	Madagascar	2008	Domestic and Wild birds	-	-	ELISA, Micro neutralization Test
Manarolla et al. (37)	Italy	2006, 2007, 2008	World birds (Owls and black birds)	-	Apathy and anorexia. Necropsy findings showed hepatomegaly, dark kidneys, hyperemic Meninges and brain	Immunohistochemistry and RT-PCR
Savini et al. (11)	Italy	2008–2009	Sentinelhorses and chickens, wild birds	-	-	ELISA, virus neutralization test, virus isolation, RT-PCR and sequencing
Savini et al. (11)	Italy	2008–2009	Wild birds		Data from the outbreak study showed birds had no remarkable gross changes at necropsy	
Weissenböck et al. (38)	Italy	1996	Wild birds (black birds)	-	Necropsy findings in wild birds revealed swollen livers and spleens, necrotizing pericloacal dermatitis,	RT-PCR and sequencing, Immunohistochemistry
Montagnaro et al. (39)	Italy	2011–2012	Hunting dogs	-	-	cELISA
Pecorari et al. (40)	Italy	2009	-	√	Presented fever and neurological symptoms	RT-PCR
Cavrini et al. (29)	Italy	2009	-	√	Detected in a patient that underwent an orthotropic liver transplant (immunocompromised). Clinical signs presented included fever, headache, skin rash, loss of neurological functions	Virus isolation, nucleic acid amplification test, heminested RT-PCR
Piero et al. (41)	Italy	2010–2011	-	√	-	Microneutralization assay
Grotolla et al. (30)	Italy	2008 and 2009; 2008 and 2012; 2010 and 2015	Wild birds	√	-	RT-PCR and sequencing, serum neutralization test
Percivalle et al. (40)	Italy	2014–205	-	√	-	Indirect immunofluorescence assay (IFA), Neutralization assay
Percivalle et al. (40)	Italy	2016–2018	-	√	-	Indirect immunofluorescence assay (IFA), Neutralization assay
Santitni et al. (31)	Croatia	2013	-	√	**Case 1** Headache, fever, somnolence, disorientation, nausea, vomiting, nuchal rigidity, intention hand tremor, hyperreflexia at all levels, extensor plantar response, dysmetria **Case 2** Headache, transient diplopia, fever, somnolence, nuchal rigidity, tongue tremor, intention hand tremor, hyperreflexia at all levels, extensor plantar response **Case 3** Headache, fever, nuchal rigidity	ELISA
Barbic et al. (42)	Croatia	2011	Horses	√	-	ELISA
Vilibic-Cavlek et al. (15)	Croatia	2018	Wild birds	√	Three human cases were detected. An immunocompromised patient aged 60 years affected with chronic lymphocytic leukemia and fatal meningoencephalitis	ELISA, RT-PCR and nucleotide sequencing
Becker et al. (28)	Germany	2011	Wild and Captive Birds	-	Apathy, staggered movements and ruffled plumage	RT-PCR and sequencing, cell culture, Immunohistochemistry
Cadar et al. (32)	Germany	2013	Bats	-	-	PCR and sequencing
Stork et al. (33)	Germany	2011–2018	Blackbirds, great gray owl, and kingfisher	-	Macroscopically, most USUV infected birds showed splenomegaly and hepatomegaly Histopathological lesions included necrosis and lymphohistiocytic inflammation within spleen, bursa fabricii, liver, heart, brain, lung and intestine	Immunohistochemistry, qRT-PCR and sequencing
Allering (34)	Germany	2012	-	√	-	Indirect immunofluorescence assay (IFA)
Cadar et al. (18)	Germany	2016	-	√	Asymptomatic	PCR, direct Sanger sequencing of the PCR amplicon
Bakonyi et al. (9)	Hungary	2005	Black Birds	-	Gross lesions observed in all cases included general congestion of internal organs, splenomegaly and hepatomegaly. Histological lesions described acute hepatitis, multiple inflammatory and necrotic foci, focal necrosis in the spleen, acute mucous enteritis, mild and focal perivascular lymphohistiocytic infiltrations in the kidney and heart, focal myocardial degeneration, vacuolar degeneration of tubular epithelial cells in the kidney, perivascular and perineuronal edema with very few lymphohistiocytic perivascular cuffs in the brain.	Reverse transcriptase-PCR, immunohistochemistry, *in situ* hybridization, viral isolation and whole genome sequencing
Bakonyi et al. (35)	Hungary	2016	Black birds	-	Same as above	Immunohistochemistry, reverse transcriptase (RT)-PCR and TaqMan real-time RT-PCR, sequencing
Nagy et al. (43)	Hungary	2018	-	√	Aseptic meningitis	Serology, Sanger sequencing
Nagy et al. (44)	Hungary	2019	-	√	5 asymptomatic blood donors	ELISA, microneutratralisation assay
Chvala et al. (45)	Austria	2001	Dead Eurasian blackbirds	-	Macroscopically hepatosplenomegaly; histologically, neuronal necrosis, myocardial lesions, and coagulative necrosis of the liver and spleen	Histopathology, immunohistochemistry, *in-situ* hybridization and reverse-transcriptase polymerase chain reaction
Chvala et al. (46)	Austria	2003–2005	Dead wild birds	-	Viral antigen distributed in the brain, spleens and hearts	Immunohistochemistry, reverse-transcriptase polymerase chain reactions. PCR amplicon sequencing
Bakonyi et al. (35)	Austria	2017	-	√	Asymptomatic, only one mentioned a stay abroad (Sicily Italy)	Usutu virus -specific RT- and RT-qPCR assays; Sanger sequencing of the amplification products
Graninger et al. (47)	Austria	2021	-	√	First reported case; Patient was presented with fever, malaise, confusion and could not respond to questions properly. Subsequently, the patient developed intermittent clonic jerks of right upper extremity USUV Europe 2 lineage was detected in the CSF and partial sequencing showed 100% identity to sequences obtained from humans, birds, and mosquitoes in the Czech Republic, Hungary, and Austria-	RT-PCR, virus neutralization test, partial genome sequencing.
Lecollinet et al. (48)	France	2015	Blackbirds	-	Gross lesions observed included hepatomegaly, splenomegaly and marked emaciation and kidney hemorrhages	Reverse transcription PCR, Virus isolation, whole genome sequencing
Simonin et al. (49)	France	2016	-	√	Atypical Neurologic Presentation; acute unilateral facial paralysis, paresthesias of both right limbs and right upper limb palsy	Reverse transcription PCR, genome sequencing
Rijks et al. (50)	The Netherlands	2016	Blackbirds' great Owls	-	Gross lesions included hepatomegaly, splenomegaly, Lung hyperemia and oedema. Histological lesions showed encephalitis, myocarditis, pneumonia, Kidney necrosis, hepatitis, splenitis, haemosiderosis and skin cloaca dermatitis	Post-mortem examination and RT-PCR
Zaaijer et al. (51)	The Netherlands	2018	-	√	No signs of infection from positive blood donors.	RT-PCR, Anti-USUV ELISA IgG assay, genome sequencing
Folly et. al. (14)	United Kingdom	2020	Eurasian blackbirds (*Turdus merula*) and one house sparrow *(Passer domesticus)*	-	Blackbirds showed marked dehydration and ataxia, and death. The house sparrow had a thin body condition and was found dead	RT-PCR, Virus isolation, Immunohistochemical detection in brain and kidney tissues

Currently, in Africa, particularly Nigeria, there are limited studies investigating the seroprevalence of USUV infection in humans. More work needs to be done to assess the risk of the virus transmission between migratory birds and competent mosquito vectors, and eventually to humans.

## USUV in other mammals

Some studies have identified USUV in other mammals such as dogs, horses, rodents, and bats (50). These findings oppose the current enzootic cycle of USUV among birds, mosquitoes. In Senegal, USUV was isolated and sequenced from samples obtained from rodents and shrews, which is the first evidence of USUV in rodents (2). Also, in Germany, USUV has been isolated from dead bats (*Pipistrellus)* mainly in the brain tissues. It is important to investigate further the contribution of bats to the transmission cycle of USUV (32). Apart from rodents, shrews, and bats, USUV infection has been detected in horses, dogs, wild boars, and wild ruminants. Antibodies specific for USUV has been identified in some horses raised in Poland, Croatia, Italy, Serbia, and Spain. (53). In addition, in 2012, USUV exposure was identified in military dogs and horses in Morocco (53). This was confirmed by virus neutralization tests. In 2014, a study also detected the presence of antibodies specific to USUV in horses in the southwestern region of Tunisia (54). In Spain, USUV-specific antibodies were also detected in wild ruminants (55). The co-infection of USUV with WNV indicates that more work needs to be done to identify which animals are truly acting as USUV reservoir hosts and which ones act as spill-over and dead-end hosts. This can help in preventing the transboundary circulation of this virus.

## The role of migratory birds in mosquito-borne virus transmission

Migratory birds are important sentinels for the introduction of some flaviviruses like WNV into multiple regions largely because the outbreak of these viruses within temperate regions normally happens in the summer or beginning of fall, which incidentally also heralds a massive number of migratory birds and vectors like mosquitoes (56). Specifically, this WNV outbreak occurred among humans living in or near wetlands where high concentrations of birds come into contact with large numbers of ornithophilic mosquitoes (57); the principal vectors from which the virus has been isolated are mainly ornithophilic mosquitoes (*Culex univittatus* in the Middle East and *C. pipiens* in Europe) (58); antibodies to the virus have been found in the blood of many migratory bird species in Eurasia (58); migratory birds have been linked with transporting related viruses in the Western Hemisphere (59); WNV has been isolated from some species of actively migrating birds e.g., the Barred Warbler [*Sylvia risoria*] in Cyprus and the Turtle Dove [*Streptopelia turtur*] in Slovakia (60); viremia sufficiently long-term to infect vector mosquitoes has been documented in several bird species (58), and migration places substantial physiologic stress on birds. For example, stress has been shown to promote immunosuppression and enhanced replication of WNV in rodents (61). Further support for the possibility that migratory birds play a major role in virus transport comes from study of related viruses. For instance, both Eastern (EEE) and Western equine encephalomyelitis alphaviruses, ecologic relatives of WNV, have been isolated from actively migrating birds in the United States (59). Evidence also indicates that the 1962 epidemic of EEE in Jamaica resulted from transport of the virus by birds from the continental United States (61). Unlike the 1999 New York City epidemic, during which large numbers of dead and dying birds, especially crows, were observed concurrently with clinical reports of human infection with the virus (62), the Old-World epidemics of WNV had few concurrent reports of deaths of infected birds (58). This difference could indicate lack of both exposure and adaptation to the virus among New World avian populations compared with Old World species. Old World data indicate that susceptibility to fatal infection with the virus varies markedly for adult and young birds, with high death rates in juveniles and high incidence of circulating antibodies in adult birds (63). Susceptibility to infection also varies considerably among species. Hooded Crows (*Corvus corone*) had both a high death rate in young birds in laboratory experiments and high levels of circulating antibodies in adults, while Rock Doves (*Columba livia*) appeared to be much less susceptible to both infection and death from the virus (63).

## Migratory birds as sentinels for USUV disease outbreak in Nigeria

**Figure 4:**
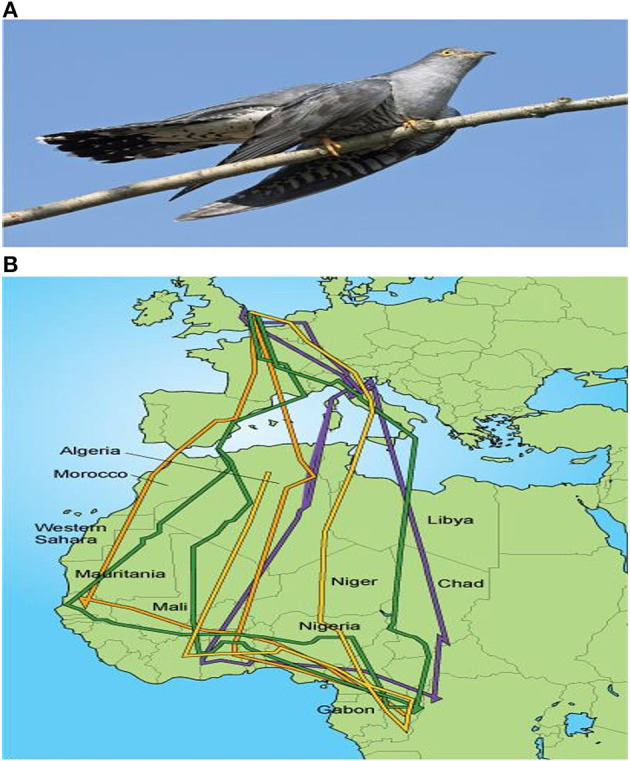
**(A)** Common cuckoo (*Cuculus canorus*), a long distance migrant that travels between the UK and Africa. **(B)** Migration paths of several cuckoos tracked from the UK to Africa and back. The multi-colored lines indicate migration paths of different individual bird species (66).

Nigeria is a West African country located within the East-Africa-Asia, the Atlantic-America, and the Black Sea/Mediterranean migratory bird flyways, some of which overlap. The dry and dusty harmattan wind flows through the Sahara in a north-easterly direction with high daytime temperatures/low humidity and cool nighttime temperatures during winter in Europe (equivalent to harmattan cold in Nigeria) (64). Innate circadian clock genes frequently control the migration of these birds throughout the winter to climatically favorable regions in the tropics. These genes may also control photoperiodic responses and the timing of life cycle events like mating and eating. These directly impact how ecosystems adapt to warmer environments and places (65). In Nigeria, birds that migrate through West Africa's sub-region and stay there are influenced by the Nigeria biotype phenology. Seasonal solid patterns are seen throughout the sizeable biological zone that runs from Siberia's Pacific coast to Western Europe's Atlantic shoreline (Figure 4). As a result, many bird species nest in this area and migrate south to spend the winter in Africa and South or Southeast Asia. These birds, originating in Europe, travel over or around the Mediterranean as they migrate to Africa (67). Nearly 2 billion songbirds, waders, birds of prey, and waterfowl each year migrate from Europe to sub-Saharan Africa, with Nigeria inclusive (68). Many of these birds during the harmattan in Nigeria undergo a north-south transatlantic migration because of the country's abundance of wetlands, rivers, natural lakes, floodplains, and dug-out dams (64).

## Zoonotic potentials oF USUV

The interaction between humans, animals, the environment, and significant health-related issues, especially the emergence and re-emergence of infectious pathogens, is not new and has been gaining global attention through active integrated, multisectoral, and multidisciplinary efforts. Likewise, the emergence and potential risk of USUV to human health are gaining scientific community recognition and entomological surveillance due to the possible impact of globalization and climate change on the genetic evolution of the virus propagation, amplification, persistence, and transmission among Culex mosquitoes; the adaptability in new hosts including humans, geographical locations and ecological niches; and capacity to be endemic in mosquito–bird life cycle and to co-circulate with WNV (19). As shown in Table 1, USUV is in countries that have reported the zoonotic possibility of the virus. Humans may get infected with USUV through *Culex* mosquito bites, and the zoonotic potential of USUV has been reported in a growing number of human cases worldwide. The zoonotic impact of USUV presents another picture in humans in Europe. Over 80 cases of sub-clinical cases of USUV infection have been reported during the surveillance of WNV, of which moderate flu-like signs were reported—rash, generalized headache, and weakness (69). The associated risk of USUV to humans was first described in Africa, where two human cases were documented in Central Africa and Burkina Faso in the 1980's and 2000's, respectively. For these two symptomatic infections, mild clinical signs involving fever, skin rashes, and jaundice, but no neurological symptoms were documented. Subsequently, newer cases have yet to be reported across Africa, which may be associated with little or no targeted surveillance programs to investigate, detect and monitor the virus and transmission dynamics among vectors, wild birds, humans, and the environment. Considering the distribution of the Culex mosquito vector in Nigeria (70) and the sub-optimal hygiene practices in most municipal slaughterhouses across the country (71), there is a possibility for the transmission of USUV from the mosquitoes and migratory birds to humans and vice versa. Although there is no substantial information from the literature if infected humans with viremia can infect the mosquito vectors in Nigeria, this could be a good project for investigation in the future. In the one health context, limited information still exists on USUV epidemiology and factors for emergence, ecology, phylogeny, and pathogenicity to human and animal health despite the intense circulation of the virus among mosquitoes and wild birds in Nigeria and many African countries (36).

## Current surveillance program for mosquito-borne viruses in Nigeria

The entomological surveillance system is a vital component of vector control since it gives valuable information on mosquito vector species, their spatio-temporal distribution, density, bionomics, and, more critically, the susceptibility and resistance of those vectors to typical insecticides used (72). The major entomological surveillance projects in Nigeria are carried out in six designated sentinel sites across five ecological zones. However, this is primarily focused on Malaria since it is the predominant mosquito-borne disease in the country (73). Currently, no standard surveillance system is enacted to prevent an outbreak of most Flaviruses, including WNV and USUV. Taking a cue from the previous outbreak and occurrences of Highly Pathogenic Avian Influenza (HPAI) in Nigeria, its association with migratory birds (74), and the devastating socio-economic impact on the poultry industry in Nigeria (75), there must be an improvement in policy formation and development of surveillance programs in Nigeria and Africa. Also, routine serological evaluation of humans living near regions where migratory birds are known to be in Nigeria would help understand the effect of the virus on humans. Significantly, proper documentation and efficient disease reporting as valuable tools in the epidemiological assessment of mosquito-borne viruses should be emphasized. It could also be helpful to incorporate screening for Flaviviruses during blood transfusions.

## Vaccines and therapeutics

Currently, there are no specific licensed vaccines and drugs for managing USUV in humans and animals. The prevention of USUV infection in humans and animals can be achieved through controlling mosquito vectors involved in the circulation of the virus. In addition, an interesting critical control point for the prevention of USUV would be the mosquito-human interface. However, there is a very low incidence of USUV infection in the human population; thus, there is little urgency to develop potent vaccines and therapeutic solutions. Many challenges are associated with developing vaccines needed to elicit protective immunity against some Flaviviral infections. There are limited models available for use in vaccine production. Adult mice serve as good models for the development of vaccines because they serve as a good platform to access immune response to immunization and complications linked to vaccination, but only suckling mice are susceptible to USUV infection, and this is a major constraint (76). To further identify more models for USUV vaccine development, a research study evaluated the effect of USUV infection in *Gallus gallus domesticus* embryonated chicken eggs. Following infection, the USUV isolate could replicate well within the chorioallantoic membrane and the allantoic fluid. Also, there was dose-dependent death of the chicken embryos after infection. This study sheds more light on the pathogenesis of USUV infection and contributes to vaccine development (69). The envelope (E), and the membrane (M) proteins are good antigenic targets for flaviviral vaccines. In the quest to develop a vaccine for USUV, a single administration of recombinant plasmid encoding the envelope (E), and the pre-membrane (M) protein was performed intramuscularly in mice. This immunization elicited a good immune response and protection against USUV challenge (77). West Nile and Usutu virus share antigenic similarity. A study checked if attenuated West Nile vaccine can induce neutralizing antibodies against USUV infection using ifnar^−/−^ mouse model. Mice vaccinated with the attenuated West Nile virus vaccine were challenged with African and European strains of USUV. The vaccinated mice exhibited lower viremia compared to unvaccinated group. This study shows that attenuated West Nile virus vaccine is protective against USUV (78). Some studies have revealed the ability of acetyl-CoA carboxylase inhibitors to block the replication of USUV in cell culture. Also, the use of drugs that prevent autophagy shows some potency against the replication of USUV *in vitro*. (12). Favipiravir, a broad-spectrum viral RNA polymerase inhibitor, was shown to reduce USUV load in a mice model. IFN-/ and IFN-receptor knockdown AG129 mice susceptible to USUV infection were used to evaluate the effect of Favipiravir on viremia caused by Usutu virus. AG129 mice were inoculated with the virus and treated with Favipiravir. The viral RNA was significantly reduced compared to the control animals. In addition, there was a reduction in the viral RNA level in body tissues. (79). Currently, more studies channeled toward vector control, discovery of vaccines, and antivirals specific for USUV infection are needed in Africa, especially Nigeria.

## Recommendations and conclusion

**Figure 5:**
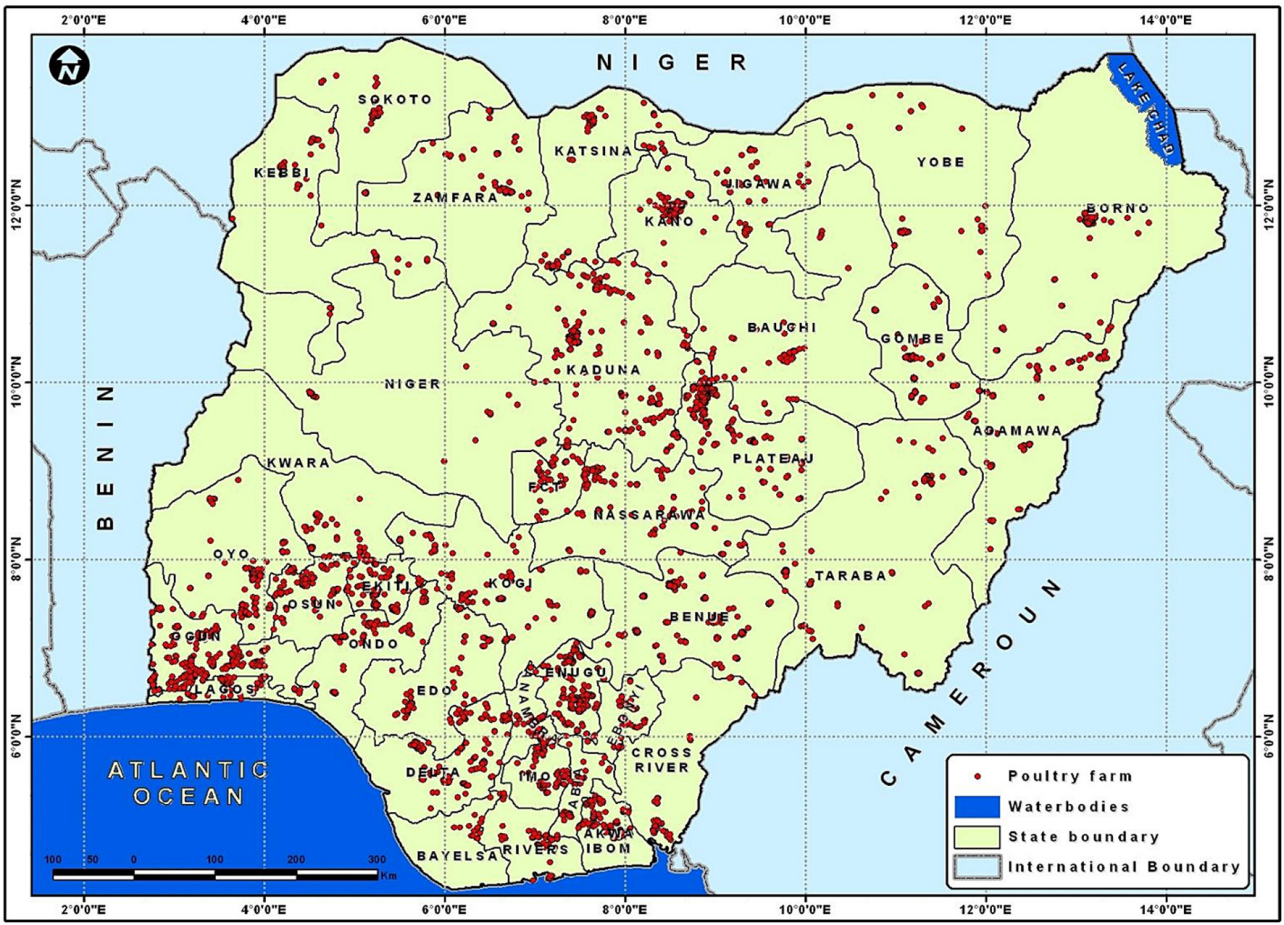
A map of Nigeria showing the distribution of poultry industry Omodele T and Okere I A (81).

The genetic variability of the USUV strains discovered in Europe, highlights the numerous importations from Africa as well as the adaptability of the strains circulating there. The complete prevalence, regional range, and seasonality of USUV infection are yet to be identified (though one could predict more cases to occur in warmer months when other mosquito-borne arboviral infections are more common) (80). Therefore, there is an urgent need for an integrated surveillance systems (“One Health”) and intelligence for emerging and re-emerging pathogens at animal, human and vectors interface to be developed and implemented in Africa and other continents to elude underreporting and underestimation of arboviral infections. A robust and responsive animal and human surveillance system should be developed and improved upon, with focus on Veterinarians and animal handlers who are constantly having interactions with wild birds and other animals. For example, sentinel chickens have been extensively used in the United States and Europe to survey for WNV. Figure 5 shows the distribution of the poultry industry in Nigeria and a cluster of this are located near the Atlantic, where some migratory birds are found in the country. The use of sentinel chickens could be an excellent tool for USUV surveillance across the six geo-political zones in Nigeria. In addition, the Nigeria government, and by extension other Africa countries, needs to build and establish facilities for confirmatory diagnosis of arboviral infections for the enhanced integrated and targeted surveillance and mitigation. For future seroprevalence studies, positive serology assays must be confirmed by several methods most especially molecular techniques (currently underutilized in Africa), and virus neutralization methods to distinguish USUV from other Flaviviruses especially WNV in order to prevent false positive or false negative conclusions, leading to underestimation of USUV infections and future negative impact on the poultry industry, animal and human populations in that clime.

## Author contributions

OCA did the conceptualization. OCA, ROA, AB, and OOA designed the table and figures. OCA, OOA, AB, SCO, IPO, ROA, and AB wrote and reviewed the draft and final versions of the manuscript. All authors contributed to the article and approved the submitted version.
